# Predicting missing links and identifying spurious links via likelihood analysis

**DOI:** 10.1038/srep22955

**Published:** 2016-03-10

**Authors:** Liming Pan, Tao Zhou, Linyuan Lü, Chin-Kun Hu

**Affiliations:** 1 Alibaba Research Center for Complexity Sciences, Alibaba Business College, Hangzhou Normal University, Hangzhou 310036, People’s Republic of China; 2 Web Sciences Center, University of Electronic Science and Technology of China, Chengdu 611731, People’s Republic of China; 3 Big Data Research Center, University of Electronic Science and Technology of China, Chengdu 611731, People’s Republic of China; 4 Institute of Physics, Academia Sinica - Nankang, Taipei 11529, Taiwan; 5 National Center for Theoretical Sciences, National Tsing Hua University, Hsinchu 30013, Taiwan; 6 Business School, University of Shanghai for Science and Technology, Shanghai 200093, China

## Abstract

Real network data is often incomplete and noisy, where link prediction algorithms and spurious link identification algorithms can be applied. Thus far, it lacks a general method to transform network organizing mechanisms to link prediction algorithms. Here we use an algorithmic framework where a network’s probability is calculated according to a predefined structural Hamiltonian that takes into account the network organizing principles, and a non-observed link is scored by the conditional probability of adding the link to the observed network. Extensive numerical simulations show that the proposed algorithm has remarkably higher accuracy than the state-of-the-art methods in uncovering missing links and identifying spurious links in many complex biological and social networks. Such method also finds applications in exploring the underlying network evolutionary mechanisms.

Link prediction algorithms aim at estimating the tendency of the existence of a link between two nodes, based on observed links, attributes of nodes, or dynamical correlations^1^,^2^,^3^. As our knowledge on many biological networks is very limited (e.g., most of the molecular interactions in cells are still unknown^4^), using predicting results to guide the laboratorial experiments rather than blindly checking all possible interactions will greatly reduce the experimental costs^5^,^6^. Besides, such predicting results for online social networks can be considered as friend recommendation^7^. Actually, how to recommend products to a target user in online e-commerce web sites is also a sub-problem of link prediction in bipartite networks where the prediction is for the target user^8^. Similar algorithms and techniques can be further applied in detecting spurious links under noisy environment^9^, in evaluating different network models by mapping evolving mechanisms into link prediction algorithms^10^, and more interestingly, in predicting the U.S. Supreme Court votes^11^.

The missing link prediction problem^1^ and the spurious link identification problem^9^ are illustrated by Figs 1 and 2, respectively, which are networks of 4 nodes. In Supplementary Fig. 1 of the Supplementary Information (SI) we give the ensemble 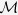 of all four-node networks. The total number of four-node networks is 2^6^ = 64, where 6 = 4 × 3/2 is the number of all possible links in the network of 4 nodes. For the missing link prediction, the task is to estimate the existence tendency of all the non-observed links based on the known network topology and nodes attributes (if we have such information). Specifically, consider an undirected network or graph *G*(*V*, *E*), where *V* is the set of 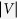 nodes and *E* is the set of 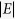 links. Multiple links and self connections are not allowed. Denoted by *U*, the universal set contains all 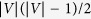 possible links. Then, the set of nonexistent links is *U* − *E*. We assume that there are some missing links (or the links that will appear in the future) in the set *U* − *E*, and the task of link prediction is to find out these links. Generally, we do not know which links are the missing or future links, otherwise we do not need to do prediction. Therefore, to test the algorithm’s accuracy, the observed links, *E*, is randomly divided into two parts: the training set, *E*^*T*^, is treated as known information, while the probe set (i.e., validation subset), *E*^*P*^, is used for testing and no information in this set is allowed to be used for prediction. Clearly, 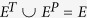 and 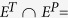. Take Fig. 1 as an example, the true network contains four nodes and four links, while the link (1, 3) is missing in the observed network *A*^*O*^. Then this missing link constitutes the probe set *E*^*P*^, and the rest observed links constitute the training set *E*^*T*^. The set of non-observed links is *U* − *E*^*T*^.

For spurious link identification, the task is to evaluate the reliability of all the observed links based on the known network topology and nodes attributes (if we have such information). Specifically, consider an undirected network *G*(*V*, *E*), where *V* is the set of nodes and *E* is the set of links. Multiple links and self connections are not allowed. Then, the set of observed links is *E*. We assume that there are some spurious links in the set *E*, and the task of spurious link identification is to find out these links. Of course, we do not know which links are the spurious link, otherwise we do not need to do identification. Therefore, to test the algorithm’s accuracy, we will randomly add some nonexistent links which will constitute the probe set *E*^*P*^, and the given network (we may say it is the true network *E*) together with the probe set constitute the training set *E*^*T*^. Clearly, *E*^*T*^ − *E*^*P*^ = *E* and 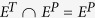. Take Fig. 2 as an example, the true network contains four nodes and four links, while the spurious link (1, 4) was added to the network to construct the training set. In reality, the training set can be considered as the real observed network which contains the errors, and the true network presented here is actually unknown for us. However, to test the algorithm’s performance we assume that the given networks are all true, otherwise we cannot make any comparison.

Traditional methods or models for predicting missing links and identifying spurious links can be roughly divided into two classes: the probabilistic models and the similarity-based algorithms: the former include the probabilistic relational model^12^, the probabilistic entity relationship model^13^, and the relational model^14^, which usually require, in addition to the observed network structure, the information about node attributes; the latter assign a similarity score to every pair of nodes and rank all non-observed links according to their scores. How to define the similarity is a nontrivial challenge: it could be simple like the common-neighbor-based indices^15^,^16^,^17^ or complicated such as random-walk-based indices^18^,^19^ and iteratively defined indices^20^,^21^.

Recently, some novel algorithms related to the likelihood analysis were proposed^9^,^22^,^23^ and shown to be more accurate than many similarity-based methods. These algorithms usually presuppose certain organizing rules of networks. In despite of detailed differences, parameters associated with the organizing rules are often learned from the observed structure and then the network ensemble is built up, accordingly, a large number of networks could be sampled out to further determine the appearing probability of each link. Representative examples include the hierarchical structure model^22^, the stochastic block model^9^ and the Kronecker graphs model^23^.

This paper introduces an algorithmic framework where a network’s probability is calculated according to a predefined structural Hamiltonian, and a non-observed link is scored by the conditional probability of adding the link to the network. The Hamiltonian is defined according to some reasonable organizing principles so that an observed network is usually of lower Hamiltonian than its randomized version. Here we consider a general principle called clustering mechanism, which declares that two nodes will have a high probability of making a link between them if they share some common neighbors or are connected by short paths. This mechanism gets direct supportive evidence from the high clustering coefficient of disparate networks^24^,^25^. In this paper, the clustering mechanism is explained as the high appearing probability of a link if its two nodes are connected by a large number of short paths and thus the corresponding Hamiltonian is defined according to the closed walk process. Numerical simulations on seven real networks showed remarkably higher accuracy of the proposed algorithm than the state-of-the-art methods in both uncovering missing links and detecting spurious links.

## Results

Common neighbor similarity performs very well in many networks^16^,^17^, indicating that the three-order loops (i.e., triangles) are preferred in the network formation. We here generalize this idea to high-order loops, and define a structural Hamiltonian:


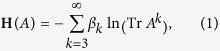


where *A* is the *N* × *N* adjacency matrix of the network with 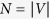 nodes, and *β*_*k*_ are the temperature parameters. When *k* > 2, the number of loops of length *k* that start and end at node *i* is 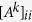. Note that, a loop is counted several times since each of its nodes can be the starting node, and given the starting node, it is counted twice by its two opposite directions. Since the loops counted here are not self-avoiding, it is more complex when a loop contains sub-loops. Roughly speaking, Tr*A*^*k*^ is 2*k* times the number of loops of length *k*, while to determine the exact number is not feasible. The approximated factor 2*k* can be taken into account by the parameter *β*_*k*_, and the cases of *k* = 1 and *k* = 2 are trivial since Tr*A*^1^ is 0 and Tr*A*^2^ is simply twice the number of total links, so we only consider the terms Tr*A*^*k*^ for *k* ≥ 3. As *k* → ∞, Tr*A*^*k*+1^/Tr*A*^*k*^ → *λ*_1_, i.e. Tr*A*^*k*^ grows exponentially with the leading eigenvalue *λ*_1_. Thus we take the logarithm to rescale each term in **H**(*A*) to the same magnitude.

For a large *k*, the increase of Tr*A*^*k*^ is simply determined by the leading eigenvalue *λ*_1_ and Tr*A*^*k*^ contains less information about the local organizations, so we introduce a cutoff *k*_*c*_. Actually, even for large networks, usually the small-world property still holds, and nodes may reach others within several steps^26^. Moreover, recent studies reveal that based only on some local information, it’s sufficient to reproduce closely many real world networks^27^. Thus a relatively small value of *k*_*c*_ is usually sufficient for many networks. How to determine *k*_*c*_ is introduced in S6 of SI, and the present results correspond to the optimal *k*_*c*_.

The structural Hamiltonian can be rewritten as:


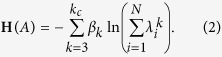


Note that we have rewritten the Hamiltonian in terms of the eigenvalues. Diagonalize the adjacency matrix as *A* = *U*^*T*^Λ*U*, where *U* is the matrix with eigenvectors in each column, and Λ the diagonal matrix of eigenvalues. Then we have 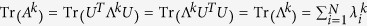. Then the Hamiltonian in equation (2) can be obtained.

Given an ensemble 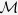, where the observed network 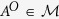 (here *A*^*O*^ = *A* − *A*^*P*^ and *A*^*P*^ is the adjacency matrix of the probe set), and the probability of the appearance of *A*^*O*^ is^28^,^29^:


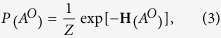


where 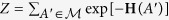 is the partition function. Such model is named exponential random graph model in social science literatures^30^. The parameters *β*_*k*_ are then chosen to maximize the probability in equation (3), see more details in Supplementary Methods.

After determining the parameters *β*_*k*_, the score of a non-observed link (*x*, *y*) ∈ *U* − *E*^*T*^ is assigned to be the conditional probability of the appearance of the link (*x*, *y*) based on the observed network:


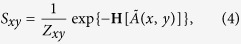


where 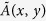 is the observed network by adding the link (*x*, *y*), and *Z*_*xy*_ is a normalization factor which defined as 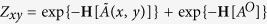. Here we assume adding the single link (*x*, *y*) to *A*^*O*^ will not largely change the topological structure and thus the parameters *β*_*k*_ for 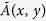 is approximately the same to those for *A*^*O*^. *S*_*xy*_ can be regarded as a kind of similarity index, so all the non-observed links will be ranked by *S*_*xy*_ for prediction: links with higher scores are more likely to exist. Obviously, the partition function *Z*_*xy*_ plays no role in producing the prediction.

In the spurious link identification problem, the score of a link (*x*, *y*) ∈ *A*^*O*^, to be spurious can be estimated by the conditional probability of the absence of this link, namely,


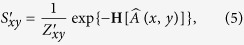


where 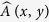 is the observed network *A*^*O*^ by removing the link (*x*, *y*), and 
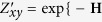

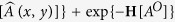
. Note that, different from the missing link prediction problem, here *A*^*O*^ = *A* + *A*^*S*^, where *A*^*S*^ is the adjacency matrix of the spurious set. Higher value of 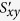 indicates a higher probability that the link (*x*, *y*) is a spurious link. The higher the value of 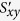, the lower reliability this link (*x*, *y*) is. A summary of notations used for the method is shown in S3 of SI.

For comparison, we introduce some benchmark methods^1^, including similarity-based algorithms and likelihood models. The simplest similarity index is the Common Neighbors (CN) index^15^, where two nodes, *x* and *y*, are more likely to have a link if they have more common neighbors, namely, 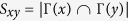, where Γ(*x*) denotes the set of neighbors of *x*. Two refined versions of CN are Adamic-Adar (AA) index^31^ 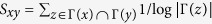, and Resource Allocation (RA) index^16^,^32^ 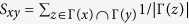. Very recently, Cannistraci, Alanis-Lobato and Ravasi^6^ simultaneously taked into account the number of common neighbors and the number of local community links (links connecting common neighbors) and proposed a series of similarity indices, including CAR, CPA, CAA, CRA and CJC indices (see details in Table 1 of ref. 6) for link prediction in brain connectomes and protein connectomes.

Different from the aforementioned local similarity indices, Katz index^33^ makes use of global topological information by summing over the collection of paths with exponentially damping according to path lengths with a parameter *α*, which reads 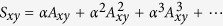, and can be rewritten in a compact form, as *S* = (*I* − *αA*)^−1^ − *I*, where *I* is the identity matrix. In our experiments, the performance of Katz index corresponds to the optimal *α*.

We also consider two likelihood models, the Hierarchical Structural Model (HSM)^22^ and the Stochastic Block Model (SBM)^9^. HSM is based on the fact that many real networks are hierarchically organized, where nodes can be divided into groups, further subdivided into groups of groups, and so forth. SBM is one of the most general network models, where nodes are partitioned into groups and the connecting probability of two nodes depends solely on the groups they belong to.

To quantify the accuracy of proposed methods, we adopt two standard metrics. The first one is called the area under the receiver operating characteristic curve (AUC value for short)^34^, which can be interpreted as the probability that a randomly chosen link in *E*^*P*^ (i.e., a missing link that indeed exists but is not observed yet) is ranked higher than a randomly chosen link in *U* − *E* (i.e., a nonexistent link). If all the link scores are generated from an independent and identical distribution, the AUC value should be about 0.5. Therefore, the degree to which the value exceeds 0.5 indicates how much the algorithm performs better than pure chance. The second one is called precision^35^, which is defined as the ratio of relevant elements to the number of selected elements. That is to say, if we take the top-*L* links as predicted links, among which *L*_*r*_ links are right (i.e., there are *L*_*r*_ links in the probe set *E*^*P*^), then the precision equals *L*_*r*_/*L*.

These two metrics can also be used to quantify the performance on detecting spurious links. In such a case, a number of spurious links are completely randomly generated that constitute the probe set *E*^*P*^ (these links are also added to *E*). In contrast to the predicting algorithm, a detecting algorithm gives an ordered list of all observed links according to their scores. The AUC value in this task becomes the probability that a randomly chosen links in *E*^*P*^ (i.e., a spurious link) is ranked lower than a randomly chosen link in *E* (i.e., an existing link). And if we pick up the last *L* links, among which *L*_*s*_ links are spurious, then the precision equals *L*_*s*_/*L*. Calculations of AUC and precision for some simple illustrative networks are given in S2 of SI.

Seven different networks from various research fields are tested. (i) Jazz^36^: The network of Jazz musicians. (ii) Metabolic^37^: The metabolic network of the nematode worm C. elegans. (iii) C. elegans^38^: The neural network of C. elegans. (iv) US Air^39^: The network of the US air transportation system. (v) FWF^40^: The food web in Florida Bay during wet season. (vi) FWM^41^: The food web in Mangrove Estuary during wet season. (vii) Macaca^42^: cortical networks of the macaque monkey. The basic topological features of such networks are summarized in Table 1. The parameters of a network include the clustering coefficient *C*^38^ and the assortative coefficient *r*^43^.

**Figure 3:**
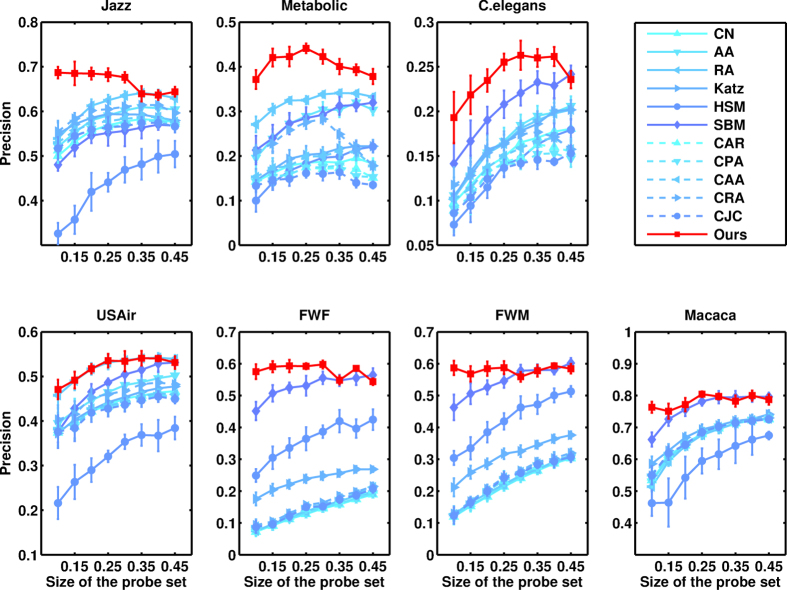
Predicting missing links for different sizes of probe set. The prediction accuracy is measured by precision.

**Figure 4:**
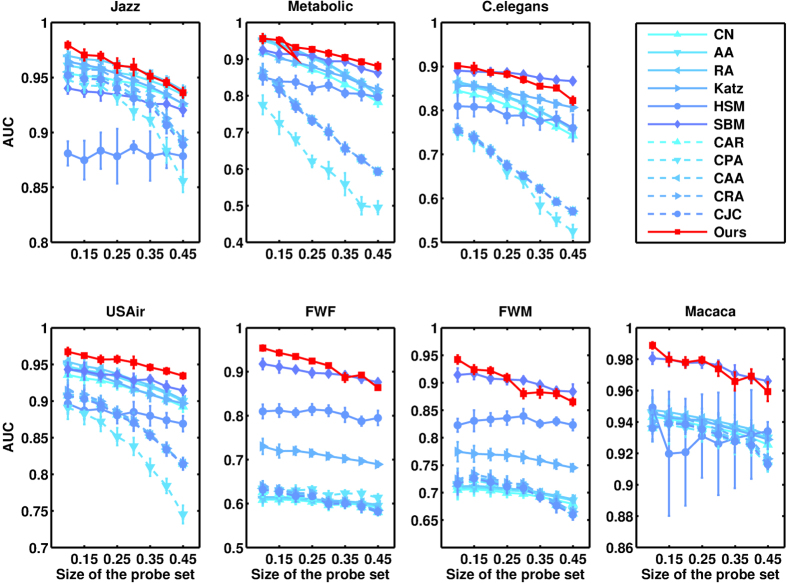
Predicting missing links for different sizes of probe set. The prediction accuracy is measured by AUC.

For each of the seven networks, the training set *E*^*T*^ contains 90% of the links, and the remaining 10% of links constitutes the probe set *E*^*P*^. To calculate precision, we set 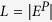, which means the number of selected elements equals the number of relevant elements. Under this specific choice of *L*, precision is equal to another metric recall that is formally defined in^35^. All the data points are obtained by averaging over 10 implementations with independently random divisions of training set and probe set. The prediction accuracies measured by precision and AUC are shown in Tables 2 and 3, respectively. For each network, the bold number in the corresponding row emphasizes the highest accuracy. Very surprisingly, for all the seven real networks, our method performs best among all state-of-the-art algorithms, usually remarkably better than the second best. The standard deviations of the prediction accuracy can be found in SI. In Figs 3 and 4, we further show that such result is not sensitive to the size of the probe set, which is the fraction of 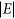 in Table 1.

We next consider the identification of spurious links, where spurious links are those links being observed but not really existent, which may be resulted from experimental errors or data noise. Prediction of missing links and identification of spurious links are considered to be equally important and highly challenging in the reconstruction of networks^9^,^44^. The framework and method proposed in this paper can also be applied to identify spurious links.

**Figure 5:**
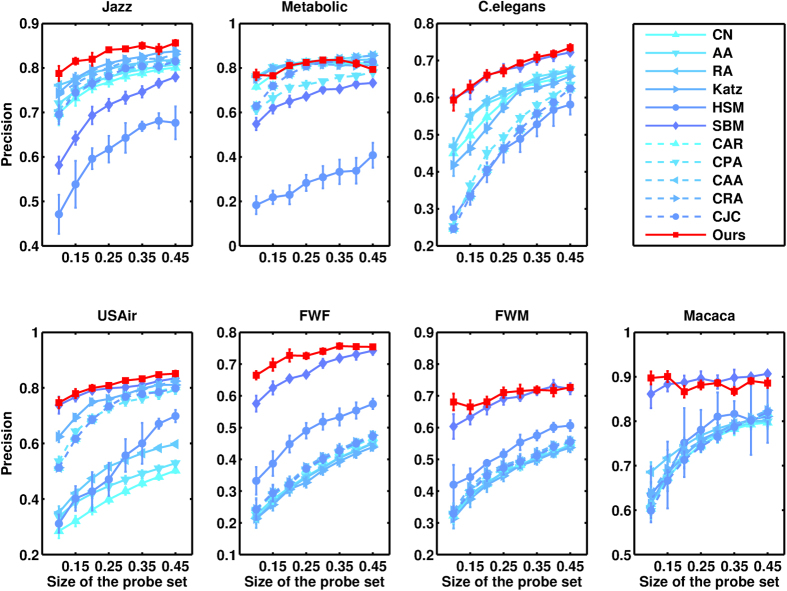
Identifying Spurious links for different sizes of probe set. The prediction accuracy is measured by precision.

**Figure 6:**
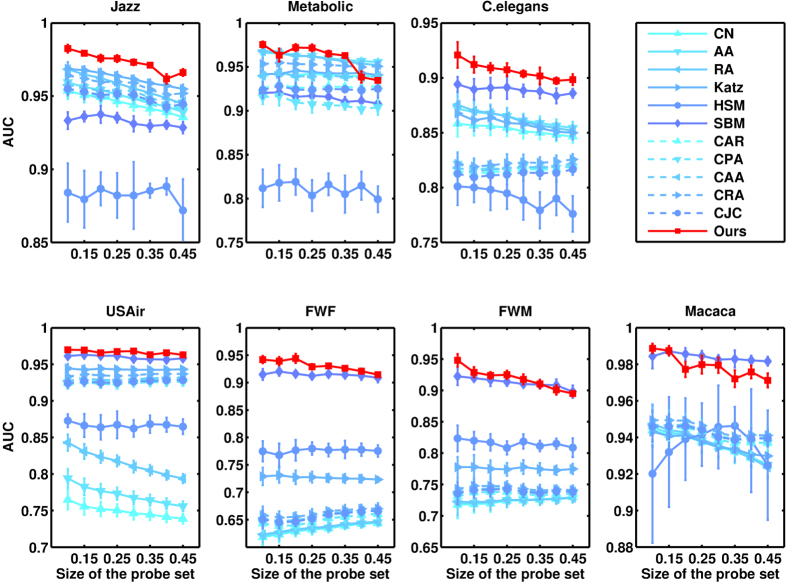
Identifying Spurious links for different sizes of probe set. The prediction accuracy is measured by AUC.

To test the validity of the algorithms, we randomly add some links to each real network, which constitute the spurious set *E*^*P*^, and the adjacency matrix of the spurious set is *A*^*S*^. Analogously, for spurious link identification, the AUC value can be interpreted as the probability that the spurious score of a randomly chosen link in *E*^*P*^ is higher than that of a randomly chosen link in *E*. The precision is defined as the ratio of the successfully identified spurious links to the top-*L* selected links with the highest spurious scores. In the experiments, we set 
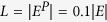
, and all the data points are averaged over 10 independent runs with different randomly generated spurious sets. The accuracies of spurious link identification measured by precision and AUC are shown in Tables 4 and 5, respectively (see SI for standard deviations). Again, our method is remarkably better than all other state-of-the-art methods and not sensitive to the size of training set, see Figs 5 and 6.

Now we apply our method to analyze the network of macaque monkey brain, where nodes are the cortical areas and links are projections between them^45^. There are three kinds of links in the focal network, namely confirmed existing, confirmed absent, and uncertain links. The reported links are based on neuroanatomical experiments, and the uncertain links are owning to conflict reports in the literature. The original network is directed, we eliminate the directions of the link by treating a link as uncertain if it is bidirectional uncertain, and as confirmed existing if it is confirmed in either direction. The undirected network consist of 32 nodes with 194 confirmed existing links, 90 confirmed absent links and 212 uncertain links. We then use our algorithm to estimate the probability of the uncertain links. To test the validity of the algorithm, we randomly hide 10% of the confirmed existing links as the probe set, and the prediction task now is to find out these hidden confirmed existing links. When calculating the prediction accuracy, we consider both the case when uncertain links are included and excluded from the candidates of missing links.

As shown in Table 6, our method can successfully find out the hidden confirmed existing links. Notably, although the number of uncertain links is much greater than confirmed absent links, there is no significant drop of the accuracy when they are included. We find that the probability that a hidden confirmed link has a higher score than an uncertain link is 0.796, indicating that uncertain links are indeed less reliable generally. Besides, we also test that the probability that an uncertain link has a higher score than a confirmed absent link is 0.634, implying that there must be some missing links in the set of uncertain links.

Now an interesting problem arises, that is among the 212 uncertain links, which are more likely to be exist. Here we use the full knowledge of the confirmed links to make predictions, see the most likely latent links predicted by our method in Table 7. ref. 42 also gave a prediction on those uncertain links. After comparison, among the top-16 predicted links shown in Table 7 there are only two links are different—PIP-V3A and PIP-V4t—which are predicted to be absent by ref. 42. While with the new progress of the studies on macaque monkey brain, the data is increasingly extended and improved^46^. The data in ref. 46 provides us an opportunity to better evaluate the algorithms. Surprisingly, in the new data set, the two controversial links are shown to be confirmed. That is to say, our structural-based method give much accurate prediction than the spatial-based method proposed in ref. 42. Besides these two links, there are three links (emphasized by bold) are also confirmed by data in ref. 46, while the other 11 predicted links are waiting for the test by real experiments in the near future.

## Discussion

Prediction is a core issue in network science, which is the only solid way to check whether our understanding of network evolution is right^10^. It covers a variety of problems, such as the prediction of missing links^1^, future links^1^, vanishing nodes^47^, reciprocal relationships^48^, spurious links^9^, and so on. In this paper, we used an algorithmic framework, where a network’s probability is estimated according to a predefined structural Hamiltonian, and the existence score of a non-observed link is quantified by the conditional probability of adding the focal link to the network while the spurious probability of an observed link is quantified by the conditional probability of deleting the link.

Since the homophily^49^ and social recommendation^50^ mechanisms ruling the real network formation both exhibit local clustering property, we define a Hamiltonian according to the closed walk process that can well take into account the structural localization. For both missing link prediction and spurious link identification, the present method performs surprisingly well, much better than all state-of-the-art methods under consideration. Notice that, although this method can find applications in small networks or some small parts (e.g., communities) of a network, it is very time-consuming and cannot be directly applied to large-scale networks. One strategy to overcome this computational limit is to use parallel algorithms. Since individual runs of the matrix diagonalization are completely independent, parallelizing the algorithm is straightforward. And also, the diagonalization of symmetric matrices itself can be parallelized^51^. In addition, we found that when estimating the model parameters, it’s not necessary to toggle all matrix elements, but roughly a 10% is sufficient to obtain the same accuracy. After determining the parameters, matrix perturbation technics can be used to compute the scores of the links. We found that by using the perturbation approximations, the algorithm still gives good predictions for many networks.

The present method can be further used to explore underlying network evolving mechanisms. For example, we can transform different evolving mechanisms into different Hamiltonians to indirectly check which mechanism could best capture the network organization principle, with a potential assumption that the mechanism corresponding to the highest link prediction accuracy is the best. We can also fix the Hamiltonian to see whether there are some sudden changes in the evolving mechanism. Such changes usually occur in technological networks such as power grid and Internet driven by the applications of some new techniques, like the new Internet Protocol for AS (autonomous system) level routers, or in online social networks according to the changes of rules and interfaces in the web sites. In S7 of SI, we show successful applications in some artificial generated networks with sudden changes in network evolution.

## Methods

Lacking an exact solution for the partition function, we apply the *maximum pseudo-likelihood method*^52^ to estimate the parameters *β*_*k*_. For any node pair (*x*, *y*), denoting *A*^*c*^(*x*, *y*) the matrix with all elements the same as *A*^*O*^ but the element 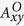 unknown, then the ratio of the conditional existence probability of the link (*x*, *y*) to the conditional nonexistence probability does not depend on the partition function, as


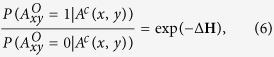


where 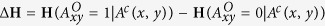. So the conditional existence probability is: 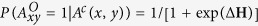.

According to the *Hammersley-Clifford Theorem*^53^ we can replace the joint likelihood of the links of the network with the product over the conditional probability of each link, given the rest of the network. Then the temperature parameters *β*_*k*_ can be estimated by maximizing the log-likelihood:


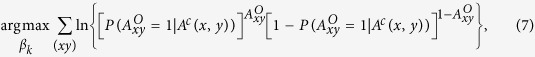


where the summation is over all node pairs. This is a convex optimization problem and we apply the gradient ascent method to estimate *β*_*k*_. Detailed steps of the parameter estimating algorithm are shown in the SI.

## Additional Information

**How to cite this article**: Pan, L. *et al.* Predicting missing links and identifying spurious links via likelihood analysis. *Sci. Rep.* **6**, 22955; doi: 10.1038/srep22955 (2016).

## Supplementary Material

Supplementary Information

## Figures and Tables

**Figure 1:**
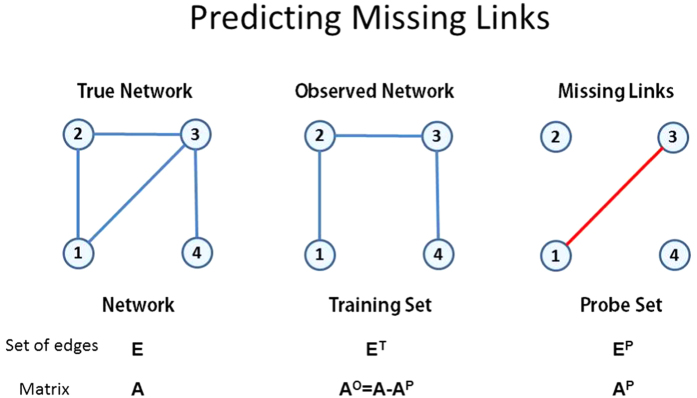
Illustrating network (graph) *G*(*V*, *E*) with 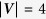 nodes and 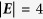 links for predicting missing links.

**Figure 2:**
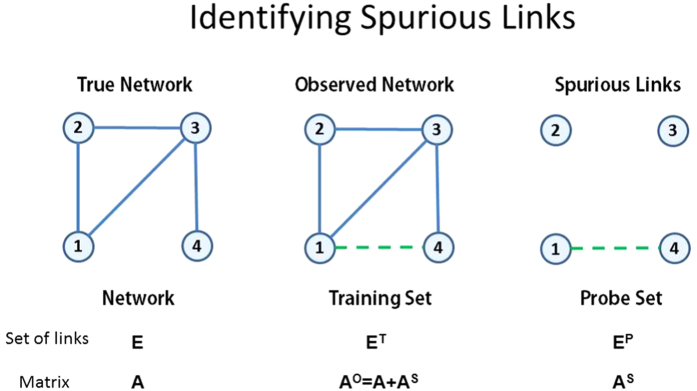
Illustrating network (graph) *G*(*V*, *E*) with 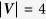 nodes and 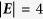 links for identifying spurious links.

**Table 1:** The basic topological features of seven real networks.

	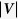	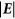	*C*	*r*	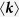	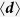	*H*
Jazz	198	2742	0.618	0.020	27.697	2.235	1.395
Metabolic	453	2025	0.647	−0.226	8.940	2.664	4.485
C. elegans	297	2148	0.292	−0.163	14.465	2.455	1.801
USAir	332	2126	0.625	−0.208	12.807	2.738	3.464
FWF	128	2075	0.335	−0.112	32.422	1.776	1.237
FWM	97	1446	0.468	−0.151	29.814	1.693	1.266
Macaca	94	1515	0.774	−0.151	32.234	1.771	1.238

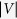
 and 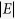 are the number of nodes and links. *C* is the clustering coefficient^38^ and *r* the assortative coefficient^43^. 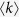 is the average degree, 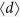 is the average shortest distance, and *H* is the degree heterogeneity, as 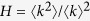.

**Table 2:** The prediction accuracy measured by precision for the seven real networks.

Precision	Ours	CN	AA	RA	Katz	HSM	SBM	CAR	CPA	CAA	CRA	CJC
Jazz	0.699	0.506	0.525	0.541	0.548	0.326	0.480	0.512	0.512	0.530	0.555	0.517
Metabolic	0.384	0.137	0.190	0.147	0.145	0.100	0.213	0.142	0.142	0.153	0.209	0.133
C. elegans	0.200	0.095	0.105	0.107	0.104	0.073	0.143	0.089	0.091	0.101	0.118	0.086
USAir	0.483	0.374	0.394	0.455	0.373	0.216	0.376	0.380	0.380	0.382	0.403	0.376
FWF	0.577	0.073	0.075	0.076	0.175	0.249	0.451	0.084	0.084	0.089	0.093	0.087
FWM	0.566	0.121	0.123	0.130	0.212	0.304	0.463	0.120	0.119	0.126	0.129	0.123
Macaca	0.755	0.528	0.533	0.513	0.586	0.462	0.662	0.543	0.542	0.551	0.549	0.550

**Table 3:** The prediction accuracy measured by AUC for the seven real networks.

AUC	Ours	CN	AA	RA	Katz	HSM	SBM	CAR	CPA	CAA	CRA	CJC
Jazz	0.981	0.955	0.962	0.971	0.964	0.881	0.940	0.952	0.948	0.955	0.961	0.952
Metabolic	0.964	0.921	0.953	0.958	0.922	0.852	0.926	0.853	0.776	0.862	0.868	0.851
C. elegans	0.909	0.847	0.863	0.867	0.856	0.810	0.889	0.756	0.749	0.757	0.760	0.754
USAir	0.972	0.935	0.946	0.952	0.943	0.896	0.942	0.907	0.890	0.909	0.914	0.906
FWF	0.949	0.610	0.611	0.614	0.738	0.809	0.917	0.625	0.633	0.633	0.638	0.631
FWM	0.942	0.709	0.712	0.715	0.774	0.822	0.914	0.710	0.711	0.718	0.723	0.715
Macaca	0.988	0.944	0.944	0.948	0.946	0.949	0.978	0.936	0.935	0.937	0.940	0.936

**Table 4:** The accuracy of spurious link identification measured by precision for the seven real networks.

Precision	Ours	CN	AA	RA	Katz	HSM	SBM	CAR	CPA	CAA	CRA	CJC
Jazz	0.794	0.701	0.723	0.761	0.745	0.471	0.582	0.697	0.690	0.703	0.731	0.695
Metabolic	0.769	0.716	0.763	0.762	0.749	0.183	0.548	0.627	0.611	0.627	0.627	0.628
C. elegans	0.593	0.446	0.465	0.465	0.433	0.277	0.599	0.243	0.253	0.243	0.243	0.246
USAir	0.749	0.642	0.686	0.686	0.626	0.311	0.738	0.513	0.536	0.513	0.513	0.513
FWF	0.672	0.232	0.229	0.218	0.220	0.342	0.575	0.239	0.241	0.243	0.246	0.242
FWM	0.657	0.322	0.328	0.332	0.313	0.420	0.603	0.333	0.323	0.331	0.332	0.329
Macaca	0.897	0.614	0.633	0.686	0.620	0.636	0.861	0.588	0.598	0.591	0.617	0.599

**Table 5:** The accuracy of spurious link identification measured by AUC for the seven real networks.

AUC	Ours	CN	AA	RA	Katz	HSM	SBM	CAR	CPA	CAA	CRA	CJC
Jazz	0.983	0.954	0.960	0.969	0.970	0.884	0.933	0.956	0.953	0.958	0.964	0.955
Metabolic	0.972	0.942	0.966	0.969	0.943	0.815	0.920	0.926	0.916	0.941	0.954	0.924
C. elegans	0.909	0.858	0.872	0.875	0.867	0.806	0.894	0.804	0.822	0.806	0.811	0.813
USAir	0.974	0.942	0.953	0.958	0.940	0.868	0.951	0.925	0.923	0.928	0.934	0.924
FWF	0.955	0.621	0.623	0.626	0.729	0.779	0.917	0.641	0.643	0.651	0.658	0.650
FWM	0.945	0.717	0.719	0.721	0.777	0.819	0.923	0.734	0.731	0.740	0.744	0.736
Macaca	0.990	0.944	0.945	0.947	0.943	0.920	0.984	0.943	0.946	0.944	0.947	0.946

**Table 6:** The accuracy of missing link prediction of the macaque brain network.

Accuracy	precision	AUC
Uncertain links excluded	0.452	0.877
Uncertain links included	0.295	0.855

**Table 7:** The 16 most likely latent links among the uncertain links and their corresponding values of the Hamiltonian.

link	Hamiltonian	link	Hamiltonian	link	Hamiltonian	link	Hamiltonian
FEF-TF	4.4565e3	PITd-PITv	4.4558e3	TF-TH	4.4553e3	V4t-LIP	4.4547e3
MSTd-MSTI	4.4565e3	V3A-VIP	4.4557e3	PIP-V3A	4.4553e3	PITd-TF	4.4546e3
CITd-CITv	4.4563e3	CITd-TF	4.4555e3	PITd-CITd	4.4550e3	PIP-V4t	4.4546e3
DP-VIP	4.4560e3	V3A-V4t	4.4555e3	PITd-TH	4.4548e3	PITv-STPp	4.4545e3
