# Supplementary material for: Predicting missing links and identifying spurious links via likelihood analysis
Source: Sci Rep. 2016 Mar 10;6:22955. doi: 10.1038/srep22955 (PMC4785364; doi:10.1038/srep22955)
Supplement: Supplementary Information [file srep22955-s1.pdf]

Supplementary Information for

Predicting Missing Links and Identifying Spurious Links via Likelihood Analysis

Liming Pan, Tao Zhou, Linyuan Lü, and Chin-Kun Hu

Contents

S1. Problem description 2
S2. Evaluation Metrics 2
A. Missing link prediction 2
B. Spurious link identification 3
S3. Table of notations 3
S4. The algorithm accuracy with standard deviations 3
S5. The algorithm for parameter estimation 4
S6. Determining the parameter k_c 6
S7. Exploring sudden change of the underlying network evolving mechanisms 7
References 9

S1. Problem description

The missing link prediction problem [1] and the spurious link identification problem [2] are described in the first three paragraphs of the main article and illustrated by Fig. 1 and Fig. 2 in the main article, respectively, which are networks of 4 nodes. The total number of networks with 4 nodes is 64 as shown in Supplementary Fig. 1 in this Supplementary Information (SI).

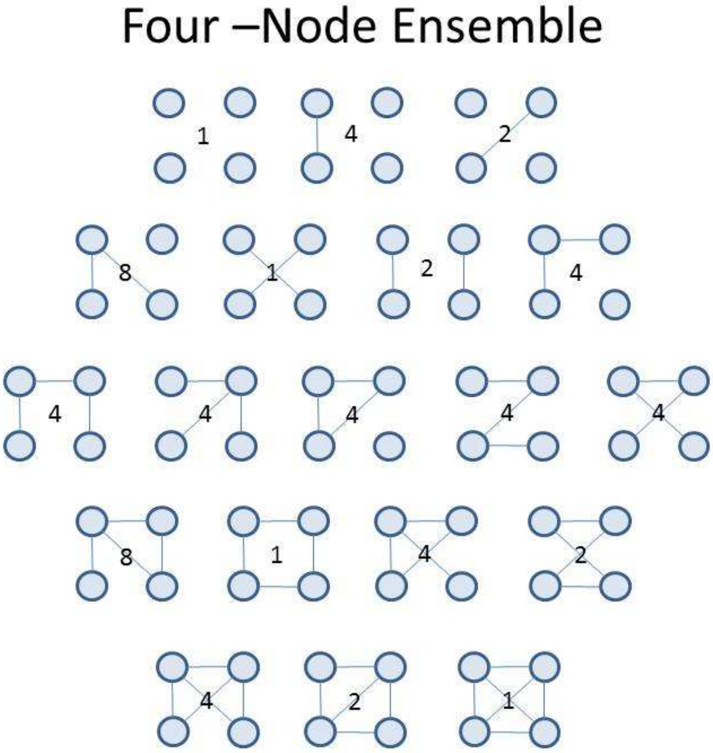

Supplementary Figure 1: The ensemble of four-node networks. The number inside each network is the total number of different networks (including the original network) that can be generated from this network via rotation and reflection along the vertical line through the center of the network. The sum of numbers inside networks in this figure is 64.

S2. Evaluation Metrics

Two standard metrics are used to quantify the accuracy of the algorithms' performances: Precision [3] and area under the receiver operating characteristic curve (AUC) [4].

A. Missing link prediction

In principle, a link prediction algorithm provides an ordered list of all non-observed links (i.e., $U - E^T$) or equivalently gives each non-observed link, say $(x, y) \in U - E^T$, a score S_{xy} to quantify its existence tendency. The AUC evaluates the algorithms performance according to the whole list while the precision only focuses on the L links with top ranks or highest scores. A detailed introduction of these two metrics is as follows.

(i) AUC: Provided the rank of all non-observed links, the AUC value can be interpreted as the probability that a randomly chosen missing link (i.e., a link in E^P) is given a higher score than a randomly chosen nonexistent link (i.e., a link in $U - E$). In the algorithmic implementation, we usually calculate the score of each non-observed link instead of giving the ordered list since the latter task is more time consuming. Then, at each time we randomly pick a missing link from E^P and a nonexistent link from $U - E$ to compare their scores, if among n independent comparisons, there are n' times the missing link having a higher score and n'' times they have the same score, the AUC value is

$$AUC = \frac{n' + 0.5n''}{n} \tag{S1}$$

If all the scores are generated from an independent and identical distribution, the AUC value should be about 0.5. Therefore, the degree to which the value exceeds 0.5 indicates how better the algorithm performs than pure chance.

(ii) Precision: Given the ranking of the non-observed links, the precision is defined as the ratio of relevant items selected to the number of items selected. That is to say, if we take the top-L links as the predicted ones, among which L_r links are right (i.e., there are L_r links in the probe set), then the precision equals L_r/L. Usually, L is no lager than $|E^P|$. An alternative metric is recall [3]. For the $|E^P|$ hidden links, if R_r of them are ranked in the top-$|E^P|$, recall is defined as $R_r/|E^P|$. So when $L = |E^P|$, precision is equal to recall. Clearly, higher precision means higher prediction accuracy.

We take Fig. 1 of the main article as an example to show how to calculate AUC and precision of predicting missing links. In this simple graph, there are four nodes, four existent links ((1, 2), (1, 3), (2, 3) and (3, 4))and two nonexistent links ((2, 4) and (1, 4)). To test the algorithms accuracy, we need to select some existent links as probe links. For instance, we pick (1, 3) as probe link (i.e., missing link), which is presented by red line in the right plot. Then, an algorithm can only make use of the information contained in the training graph (see the middle plot). Common neighbor algorithm assigns scores of all non-observed links as $S_{13} = 1$, $S_{24} = 1$, $S_{14} = 0$. To calculate AUC, we need to compare the scores of a probe link and a nonexistent link. There are two pairs in total: $S_{13} = S_{24}$ and $S_{13} > S_{14}$. Hence, the AUC value equals $(1 \times 1 + 1 \times 0.5)/2 = 0.75$. For precision, if $L = 1$, the predicted link is either (1, 3) or (2, 4). Clearly, the former is right while the latter is wrong, and thus the precision equals 0.5. For more detail information on link prediction problem, please see the review article ref. [1].

B. Spurious link identification

Similarly, we also use AUC and precision to evaluate the algorithm's performance on spurious link identification. In such case, a number of spurious links are completely randomly generated to constitute the probe set E^P presented by adjacency matrix A^S. Now the observed network is $A^O = A + A^S$. In contrast to the predicting algorithm, a detecting algorithm gives an ordered list of all observed links $(E + E^P)$ according to their scores of existing. The AUC value in this task becomes the probability that a randomly chosen links in E^P (i.e., a spurious link) is ranked lower than a randomly chosen link in E (i.e., an existing link). At each time we randomly pick a spurious link and a existent link to compare their scores, if among n independent comparisons, there are n' times the spurious link having a lower score and n'' times they have the same score, the AUC value is

$$AUC = \frac{n' + 0.5n''}{n} \tag{S2}$$

And if we pick up the last L links, among which L_s links are spurious (i.e., there are L_s links in the set E^P), then the precision equals L_s/L.

We take Fig. 2 of the main article as an example to show how to calculate AUC and precision of identifying spurious links. In this simple graph, there are four nodes, four existent links ((1, 2), (1, 3), (2, 3) and (3, 4)) and two nonexistent links ((2, 4) and (1, 4)). To test the algorithm's accuracy, we randomly add one spurious link, say (1, 4), as probe link which is presented by green dashed line in the right plot. Then, an algorithm can only make use of the information contained in the training graph (i.e., $E^T = E + E^P$, see the middle plot). Common neighbor algorithm assigns scores of all observed links as $S'_{12} = 1$, $S'_{13} = 2$, $S'_{14} = 1$, $S'_{23} = 1$ and $S'_{34} = 1$. To calculate AUC, we need to compare the scores of a probe link and a existent link. There are four pairs in total: $S'_{14} = S'_{12}$, $S'_{14} < S'_{13}$, $S'_{14} = S'_{23}$ and $S'_{14} = S'_{34}$. Hence, the AUC value equals $(1 \times 1 + 3 \times 0.5)/4 = 0.625$. For precision, if $L = 1$, the predicted link can be any one among (1, 2) (1, 4), (2, 3) and (3, 4). Clearly, only (1, 4) is right, and thus the precision equals 0.25.

S3. Table of notations

The definition of some notations used in this paper are summarized in Supplementary Table. 1.

S4. The algorithm accuracy with standard deviations

The standard deviations of algorithm accuracy are shown in Supplementary Table 2 and Supplementary Table 3 for predicting missing links on seven networks, and in Supplementary Table 4 and Supplementary Table 5 for spurious link identification on seven networks. Each number is obtained by averaging over ten independent implements. In each Table, the upper part is about the comparison of our approach with CN, AA, RA, Katz, HSM, and SBM approaches defined in the main article, and the lower part is about comparison of our approach with CAR, CPA, CAA, CRA, and CJC defined in Table 1 of [5].

Notations	Description
A	The adjacency matrix of the true network
A^O	The adjacency matrix of the observed network
A^P	The adjacency matrix of the missing links
A^S	The adjacency matrix of the spurious links
E	The set of edges of the true network
E^T	The training set, the set of edges of the observed network
E^P	The probe set, the missing or spurious links
$A^c(x,y)$	$A^c(x,y)$ is identical to A^O only with the link (x,y) unknown
$\tilde{A}(x,y)$	The adjacency matrix by adding the link (x,y) to A^O
$\widehat{A}(x,y)$	The adjacency matrix by deleting the link (x,y) from A^O
S_{xy}	The conditional probability of a node pair (x,y) to be a latent link
S'_{xy}	The conditional probability of a node pair (x,y) to be a spurious link

Supplementary Table 1: Some of the notations used in the Letter.

Precision	Ours	CN	AA	RA	Katz	HSM	SBM
Jazz	$\mathbf{0.699 \pm 0.024}$	0.506 ± 0.026	0.525 ± 0.025	0.541 ± 0.024	0.548 ± 0.020	0.326 ± 0.024	0.480 ± 0.014
Metabolic	$\mathbf{0.384 \pm 0.015}$	0.137 ± 0.025	0.190 ± 0.023	0.267 ± 0.026	0.145 ± 0.026	0.100 ± 0.025	0.213 ± 0.032
C.elegans	$\mathbf{0.200 \pm 0.027}$	0.095 ± 0.018	0.105 ± 0.017	0.104 ± 0.018	0.104 ± 0.018	0.073 ± 0.012	0.143 ± 0.020
USAir	$\mathbf{0.483 \pm 0.043}$	0.374 ± 0.030	0.394 ± 0.030	0.455 ± 0.030	0.373 ± 0.031	0.216 ± 0.036	0.376 ± 0.024
FWF	$\mathbf{0.577 \pm 0.018}$	0.073 ± 0.014	0.075 ± 0.014	0.076 ± 0.015	0.175 ± 0.021	0.249 ± 0.020	0.451 ± 0.029
FWM	$\mathbf{0.566 \pm 0.025}$	0.121 ± 0.021	0.123 ± 0.021	0.130 ± 0.022	0.212 ± 0.028	0.304 ± 0.022	0.463 ± 0.040
Macaca	$\mathbf{0.755 \pm 0.020}$	0.528 ± 0.024	0.533 ± 0.022	0.513 ± 0.021	0.586 ± 0.023	0.462 ± 0.040	0.662 ± 0.022

Precision	Ours	CAR	CPA	CAA	CRA	CJC
Jazz	$\mathbf{0.699 \pm 0.024}$	0.512 ± 0.027	0.512 ± 0.028	0.530 ± 0.029	0.555 ± 0.024	0.517 ± 0.025
Metabolic	$\mathbf{0.384 \pm 0.015}$	0.142 ± 0.013	0.142 ± 0.012	0.153 ± 0.013	0.209 ± 0.016	0.133 ± 0.012
C.elegans	$\mathbf{0.200 \pm 0.027}$	0.089 ± 0.024	0.091 ± 0.024	0.101 ± 0.025	0.118 ± 0.020	0.086 ± 0.025
USAir	$\mathbf{0.483 \pm 0.043}$	0.380 ± 0.037	0.380 ± 0.037	0.382 ± 0.037	0.403 ± 0.032	0.376 ± 0.037
FWF	$\mathbf{0.577 \pm 0.018}$	0.084 ± 0.020	0.084 ± 0.020	0.089 ± 0.019	0.093 ± 0.018	0.087 ± 0.018
FWM	$\mathbf{0.566 \pm 0.025}$	0.120 ± 0.024	0.119 ± 0.024	0.126 ± 0.027	0.129 ± 0.022	0.123 ± 0.026
Macaca	$\mathbf{0.755 \pm 0.020}$	0.543 ± 0.030	0.542 ± 0.030	0.551 ± 0.026	0.549 ± 0.026	0.550 ± 0.029

Supplementary Table 2: The prediction accuracy measured by precision for the seven real networks. Upper part: comparison of our approach with CN, AA, RA, Katz, HSM, and SBM; lower part: comparison of our approach with CAR, CPA, CAA, CRA, and CJC.

S5. The algorithm for parameter estimation

Instead of maximize the entire probability of the observed network, we maximize the logarithm of the product over the marginal probability of each link:

$$P(A^O \mid \beta_k) = \sum_{(xy)} \ln \left\{ \Big[P\big(A^O_{xy} = 1 \mid A^c(x,y)\big) \Big]^{A^O_{xy}} \times \Big[1 - P\big(A^O_{xy} = 1 \mid A^c(x,y)\big) \Big]^{1 - A^O_{xy}} \right\}, \tag{S3}$$

here

$$P\left(A^O_{xy} = 1 \mid A^c(x,y)\right) = \frac{1}{1 + \exp(\Delta\mathbf{H})}, \tag{S4}$$

and

$$\Delta\mathbf{H} = \mathbf{H}(A^O_{xy} = 1 \mid A^c(x,y)) - \mathbf{H}(A^O_{xy} = 0 \mid A^c(x,y)). \tag{S5}$$

AUC	Ours	CN	AA	RA	Katz	HSM	SBM
Jazz	**0.981 ± 0.004**	0.955 ± 0.005	0.962 ± 0.005	0.971 ± 0.004	0.964 ± 0.005	0.881 ± 0.011	0.940 ± 0.005
Metabolic	**0.964 ± 0.009**	0.921 ± 0.012	0.953 ± 0.011	0.958 ± 0.010	0.922 ± 0.013	0.852 ± 0.021	0.926 ± 0.009
C.elegans	**0.909 ± 0.012**	0.847 ± 0.014	0.863 ± 0.015	0.867 ± 0.014	0.856 ± 0.013	0.810 ± 0.027	0.889 ± 0.007
USAir	**0.972 ± 0.008**	0.935 ± 0.011	0.946 ± 0.010	0.952 ± 0.010	0.943 ± 0.009	0.896 ± 0.014	0.942 ± 0.009
FWF	**0.949 ± 0.004**	0.610 ± 0.015	0.611 ± 0.015	0.614 ± 0.015	0.738 ± 0.010	0.809 ± 0.016	0.917 ± 0.012
FWM	**0.942 ± 0.009**	0.709 ± 0.017	0.712 ± 0.016	0.715 ± 0.017	0.774 ± 0.018	0.822 ± 0.012	0.914 ± 0.012
Macaca	**0.988 ± 0.002**	0.944 ± 0.010	0.944 ± 0.010	0.948 ± 0.008	0.946 ± 0.009	0.949 ± 0.011	0.978 ± 0.003

AUC	Ours	CAR	CPA	CAA	CRA	CJC
Jazz	**0.981 ± 0.004**	0.952 ± 0.008	0.948 ± 0.010	0.955 ± 0.008	0.961 ± 0.008	0.952 ± 0.008
Metabolic	**0.964 ± 0.009**	0.853 ± 0.021	0.776 ± 0.026	0.862 ± 0.020	0.868 ± 0.021	0.851 ± 0.019
C.elegans	**0.909 ± 0.012**	0.756 ± 0.010	0.749 ± 0.015	0.757 ± 0.010	0.760 ± 0.010	0.754 ± 0.010
USAir	**0.972 ± 0.008**	0.907 ± 0.015	0.890 ± 0.016	0.909 ± 0.015	0.914 ± 0.015	0.906 ± 0.015
FWF	**0.949 ± 0.004**	0.625 ± 0.011	0.633 ± 0.013	0.633 ± 0.013	0.638 ± 0.012	0.631 ± 0.012
FWM	**0.942 ± 0.009**	0.710 ± 0.020	0.711 ± 0.020	0.718 ± 0.020	0.723 ± 0.020	0.715 ± 0.020
Macaca	**0.988 ± 0.002**	0.936 ± 0.008	0.935 ± 0.008	0.937±0.008	0.940 ± 0.008	0.936 ± 0.008

Supplementary Table 3: The prediction accuracy measured by AUC for the seven real networks. Upper part: comparison of our approach with CN, AA, RA, Katz, HSM, and SBM; lower part: comparison of our approach with CAR, CPA, CAA, CRA, and CJC.

Precision	Ours	CN	AA	RA	Katz	HSM	SBM
Jazz	**0.794 ± 0.015**	0.701 ± 0.014	0.723 ± 0.014	0.761 ± 0.015	0.745 ± 0.016	0.471 ± 0.044	0.582 ± 0.022
Metabolic	**0.769 ± 0.017**	0.716 ± 0.013	0.763 ± 0.012	0.762 ± 0.012	0.749 ± 0.015	0.183 ± 0.040	0.548 ± 0.028
C.elegans	0.593 ± 0.029	0.446 ± 0.023	0.465 ± 0.019	0.465 ± 0.019	0.433 ± 0.034	0.277 ± 0.029	**0.599 ± 0.020**
USAir	**0.749 ± 0.023**	0.642 ± 0.021	0.686 ± 0.019	0.686 ± 0.019	0.626 ± 0.020	0.311 ± 0.033	0.738 ± 0.030
FWF	**0.672 ± 0.018**	0.232 ± 0.023	0.229 ± 0.024	0.218 ± 0.023	0.220 ± 0.024	0.342 ± 0.046	0.575 ± 0.025
FWM	**0.657 ± 0.021**	0.322 ± 0.026	0.328 ± 0.029	0.332 ± 0.031	0.313 ± 0.031	0.420 ± 0.062	0.603 ± 0.040
Macaca	**0.897 ± 0.016**	0.614 ± 0.027	0.633 ± 0.029	0.686 ± 0.030	0.620 ± 0.030	0.636 ± 0.062	0.861 ± 0.031

Precision	Ours	CAR	CPA	CAA	CRA	CJC
Jazz	**0.794 ± 0.015**	0.697 ± 0.020	0.690 ± 0.020	0.703 ± 0.021	0.731 ± 0.018	0.695 ± 0.021
Metabolic	**0.769 ± 0.017**	0.627 ± 0.005	0.611 ± 0.013	0.627 ± 0.005	0.627 ± 0.005	0.628 ± 0.007
C.elegans	0.593 ± 0.029	0.243 ± 0.006	0.253 ± 0.012	0.243 ± 0.006	0.243 ± 0.006	0.246 ± 0.007
USAir	**0.749 ± 0.023**	0.513 ± 0.008	0.536 ± 0.018	0.513 ± 0.008	0.513 ± 0.008	0.512 ± 0.008
FWF	**0.672 ± 0.018**	0.239 ± 0.019	0.241 ± 0.035	0.243 ± 0.019	0.246 ± 0.018	0.242 ± 0.032
FWM	**0.657 ± 0.021**	0.333 ± 0.029	0.323 ± 0.023	0.331 ± 0.032	0.332 ± 0.028	0.329 ± 0.025
Macaca	**0.897 ± 0.016**	0.588 ± 0.023	0.598 ± 0.018	0.591 ± 0.026	0.617 ± 0.023	0.599 ± 0.017

Supplementary Table 4: The accuracy of spurious link identification measured by precision for the seven real networks. Upper part: comparison of our approach with CN, AA, RA, Katz, HSM, and SBM; lower part: comparison of our approach with CAR, CPA, CAA, CRA, and CJC.

Taking the gradient of the likelihood function with respect to β_k, we obtain

$$\nabla P(A^O \mid \beta_k) = \sum_{(xy)} \Big[A^O_{xy} - P\big(A^O_{xy} = 1 \mid A^c(x,y)\big)\Big]\phi^k_{(xy)}, \tag{S6}$$

where $\phi^k_{(xy)}$ is the statistical difference of the kth term of the Hamiltonian when A^O_{xy} is toggled from 1 to 0 while holding the rest of the observed network unchanged. Now the parameters can be updated using the gradient ascent rule:

$$\beta_k := \beta_k + \lambda \nabla P(A^O \mid \beta_k), \tag{S7}$$

where λ controls the updating rate. The steps of the algorithm are listed as follows:

AUC	Ours	CN	AA	RA	Katz	HSM	SBM
Jazz	$\mathbf{0.983 \pm 0.002}$	0.954 ± 0.005	0.960 ± 0.005	0.969 ± 0.004	0.970 ± 0.004	0.884 ± 0.020	0.933 ± 0.006
Metabolic	$\mathbf{0.972 \pm 0.005}$	0.942 ± 0.009	0.966 ± 0.006	0.969 ± 0.006	0.943 ± 0.010	0.815 ± 0.025	0.920 ± 0.009
C.elegans	$\mathbf{0.909 \pm 0.010}$	0.858 ± 0.011	0.872 ± 0.011	0.875 ± 0.012	0.867 ± 0.010	0.806 ± 0.021	0.894 ± 0.006
USAir	$\mathbf{0.974 \pm 0.003}$	0.942 ± 0.006	0.953 ± 0.006	0.958 ± 0.005	0.940 ± 0.007	0.868 ± 0.020	0.951 ± 0.006
FWF	$\mathbf{0.955 \pm 0.005}$	0.621 ± 0.016	0.623 ± 0.016	0.626 ± 0.015	0.729 ± 0.016	0.779 ± 0.025	0.917 ± 0.007
FWM	$\mathbf{0.945 \pm 0.007}$	0.717 ± 0.020	0.719 ± 0.020	0.721 ± 0.020	0.777 ± 0.016	0.819 ± 0.021	0.923 ± 0.014
Macaca	$\mathbf{0.990 \pm 0.003}$	0.944 ± 0.009	0.945 ± 0.009	0.947 ± 0.010	0.943 ± 0.009	0.920 ± 0.038	0.984 ± 0.006

AUC	Ours	CAR	CPA	CAA	CRA	CJC
Jazz	$\mathbf{0.983 \pm 0.002}$	0.956 ± 0.007	0.953 ± 0.005	0.958 ± 0.007	0.964 ± 0.007	0.955 ± 0.004
Metabolic	$\mathbf{0.972 \pm 0.005}$	0.926 ± 0.010	0.916 ± 0.008	0.941 ± 0.009	0.954 ± 0.007	0.924 ± 0.009
C.elegans	$\mathbf{0.909 \pm 0.010}$	0.804 ± 0.011	0.822 ± 0.011	0.806 ± 0.011	0.811 ± 0.011	0.813 ± 0.011
USAir	$\mathbf{0.974 \pm 0.003}$	0.925 ± 0.004	0.923 ± 0.006	0.928 ± 0.004	0.934 ± 0.004	0.924 ± 0.006
FWF	$\mathbf{0.955 \pm 0.005}$	0.641 ± 0.021	0.643 ± 0.016	0.651 ± 0.020	0.658 ± 0.020	0.650 ± 0.016
FWM	$\mathbf{0.945 \pm 0.007}$	0.734 ± 0.024	0.731 ± 0.018	0.740 ± 0.024	0.744 ± 0.024	0.736 ± 0.018
Macaca	$\mathbf{0.990 \pm 0.003}$	0.943 ± 0.006	0.946 ± 0.008	0.944 ± 0.006	0.947 ± 0.006	0.946 ± 0.008

Supplementary Table 5: The accuracy of spurious link identification measured by AUC for the seven real networks. Upper part: comparison of our approach with CN, AA, RA, Katz, HSM, and SBM; lower part: comparison of our approach with CAR, CPA, CAA, CRA, and CJC.

(i) For all node pairs (xy), calculate $\phi_{(xy)}^{k}$, which is defined as the statistical difference of the kth term of the Hamiltonian when A_{xy}^{O} is toggled from 1 to 0 while the rest of the observed network remains static.

(ii) Initial the parameters β_k, and update according equation (S7), till convergence.

S6.Determining the parameter k_c

Empirically each network has a specific optimal k_c^* which depends on the structure of the network intricately, and it is difficult to get an analytical solution on k_c^*, so that we can only numerically estimate the optimal k_c^* based on the observed structure (E^T). In our experiments, we know exactly the probe set, therefore the optimal k_c^* subject to the highest accuracy can be obtain via varying k_c in the experiments. Supplementary Fig. 2 shows the dependence of prediction accuracy on the cutoff k_c. We name this k_c^* as true value. However, in practice we don't know what are the missing links or future links, what we can use is just the observed information. Therefore, a realistic way is to learn the optimal k_c^* based on the observed network, namely A^O, and this k_c^* will be further applied to predict missing links or identifying spurious links of A^O. Specifically, for missing link prediction, after the network is divided into the training set (i.e., E^T contains $0.9|E|$ links) and the probe set (i.e., E^P contains $0.1|E|$ links), we will learn k_c^* by proceeding the link prediction algorithm on training set. Firstly, we hide another set B consists of $0.1|E|$ links from the training set. Then we use the remaining $0.8|E|$ links in training set (denote by set D) to predict the newly hidden links in B under different k_c. Finally, the k_c^* will be obtained when the highest accuracy is reached. The results in Tables 2 and 3 in the main text are obtained by using the training set E^T to predict the links in probe set E^P under k_c^*. Here we assume that k_c^* calculated from Case 1 (D as training set and B as probe set) and Case 2 (E^T as training set and E^P as probe set) are approximately the same to each other. In real applications, such a procedure can be used to choose the k_c^* with calculating from known links.

For spurious link identification, the procedure is almost the same. By adding the probe set E^P to the network, we obtain the training set E^T. Then a fraction of $0.1|E|$ links from E^T will be hidden as set B, and then the remaining links constitute set D. Similar to the case for predicting missing link, here we firstly use D to predicting missing links in B under different k_c, and the k_c^* is obtained subject to the highest accuracy. The results in Tables 4 and 5 in the main text are obtained by identifying the spurious links in probe set E^P based on the training set E^T under k_c^*. Note that k_c^* for precision and AUC are not always consistent, in this Letter we also choose k_c^* for precision to present the results of AUC. The approximate k_c^* for precision obtained by the above described procedure and the true optimal k_c^* (as indicated in Supplementary Fig. 2) are shown in Supplementary Table 6 for comparison. It can be found that the value of the approximate k_c^* as well as its performance are all near the true optimal cases.

To estimate the optimal k_c^* will result in additional computational costs, however, as shown in Supplementary Fig. 2, except for too small or too large k_c, the algorithms performance varies slightly for a considerable range of k_c.

For example, if we fix $k_c = 8$ for every network, we can get almost the same prediction accuracy as the optimal one shown in Table 2 to Table 5 in the main manuscript. Unlucky, we cannot obtain the tendency of optimal k_c^* versus network size, since the present method is very time-consuming. In a word, this is indeed a limitation, but may be not a very critical problem in the practical application.

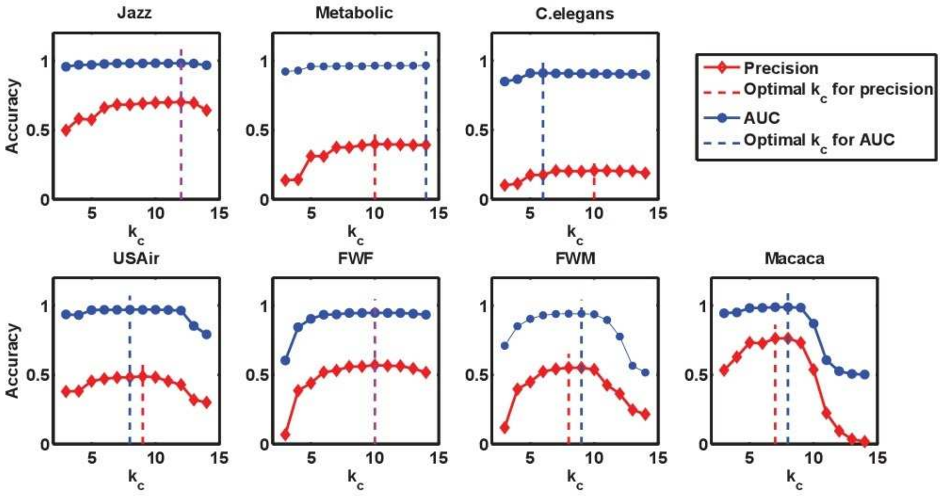

Supplementary Figure 2: The prediction accuracy versus the parameter k_c for the seven real networks, with red and blue curves standing for precision and AUC, respectively. The optimal parameter is denoted by a vertical dashed line with the corresponding color. Note that the optimal line is drawn in purple when the two optimal values coincide.

Net	Jazz	Metabolic	C.elegans	USAir	FWF	FWM	Macaca
True k_c^*	12	10	10	9	10	8	7
MLP k_c^*	11	14	7	6	11	9	7
SLI k_c^*	10	10	8	8	12	9	7

Supplementary Table 6: The value of k_c^* for seven different networks. The three rows of data correspond to the true value of k_c^*, the approximate k_c^* for missing link prediction (MLP) and the approximate k_c^* for spurious link identification (SLI), respectively.

S7. Exploring sudden change of the network evolving mechanisms

The present method can be used to explore underlying network evolving mechanisms. For example, we can transform different evolving mechanisms into different Hamiltonians to indirectly check which mechanism could best capture the network organization principle, with a potential assumption that the mechanism corresponding to the highest link prediction accuracy is the best. We can also fix the Hamiltonian to see whether there are some sudden changes in the evolving mechanism. Such changes usually occur in technological networks such as power grid and Internet for some new techniques, like the new Internet Protocol for AS (autonomous system) level routers, or in online social networks according to the changes of rules and interfaces in the web sites. Further information about AS can be found at http://en.wikipedia.org/wiki/Autonomous_System_(Internet).

Here we show one example as follows. We consider three different mechanisms: (1) Transitivity, where new links are added between node x and node y with probability $p_{xy} \propto |\Gamma_x \bigcap \Gamma_y|$; (2) Preferential Attachment (PA), with probability $p_{xy} \propto k_x k_y$; and (3) Erdos-Renyi (ER) Rule, where new links are added completely randomly. Initially the network is prepared as an Erdos-Renyi network with $N = 100$ nodes and connecting probability $p = 0.08$. At each step, 50 new links are added to the network. For time steps $T = 1, 2, 3, 4, 5$, the network is growing under a specific mechanism, and at the step $T = 6$, it's suddenly switched to another mechanism. At each time step T, the parameters β_k are estimated according to the network at time step $T - 1$, and the AUC value of the newly added links at step T is calculated according to the Hamiltonian for the network at step $T - 1$. As shown in Supplementary

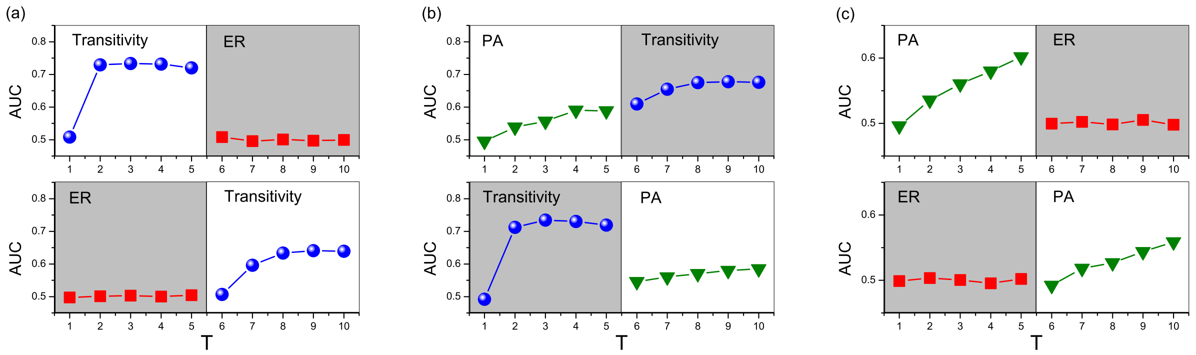

Supplementary Figure 3: Exploring sudden changes of the network evolving mechanism: (a) Transitivity to ER (top) and ER to Transitivity (bottom), (b) PA to Transitivity (top) and Transitivity to PA (bottom), and (c) PA to ER (top) and ER to PA (bottom). Each data point is averaged over 100 independent runs.

Fig. 3, there are six different cases for sudden switch between two mechanisms, and for each of them, the present method is able to capture the jump at $T = 6$.

From above numerical experiments, we can see that our method can successfully detect the sudden change points of the network evolving mechanism. Since the Hamiltonian is a comprehensive description of the network, the method does not require any prior knowledge about the mechanism itself.

[1] Lü, L. & Zhou, T. Link prediction in complex networks: A survey. *Physica A* **390**, 1150 (2011).
[2] Guimerà R. & Sales-Pardo, M. Missing and spurious interactions and the reconstruction of complex networks. *Proc. Acad. Natl. Sci. U.S.A.* **106**, 22073 (2009).
[3] Herlocker, J. L., Konstann, J. A., Terveen, K. & Riedl, J. T. Evaluating collaborative filtering recommender systems. *ACM Trans. Inf. Syst.* **22**, 5 (2004).
[4] Hanely, J. A. & McNeil, B. J. The meaning and use of the area under a receiver operating characteristic (ROC) curve. *Radiology* **143**, 29 (1982).
[5] Cannistraci, C. V., Alanis-Lobato, G. & Ravasi, T. From link-prediction in brain connectomes and protein interactomes to the local-community-paradigm in complexnetworks. *Sci. Rep.* **3**, 1613 (2013).
[6] Barabási, A.-L. & Albert, R. Emergence of scaling in random networks. *Science* **286**, 509 (1999).
[7] Erdös, P. & Rényi, A. On random graphs. *Publicationes Mathematicae* **6**, 290 (1959).
